# Molecular methods for tracking residual *Plasmodium falciparum* transmission in a close-to-elimination setting in Zanzibar

**DOI:** 10.1186/s12936-020-3127-x

**Published:** 2020-01-29

**Authors:** Benjamin Grossenbacher, Aurel Holzschuh, Natalie E. Hofmann, Kali Abdullah Omar, Logan Stuck, Bakar Shariff Fakih, Abdullah Ali, Joshua Yukich, Manuel W. Hetzel, Ingrid Felger

**Affiliations:** 1 Swiss Tropical and Public Health Institute, Socinstrasse 57, 4051 Basel, Switzerland; 2 University of Basel, Basel, Switzerland; 3 Zanzibar Malaria Elimination Programme, Ministry of Health, Zanzibar, United Republic of Tanzania; 4 School of Public Health and Tropical Medicine, Tulane University, New Orleans, USA; 5 Ifakara Health Institute, Dar es Salaam, United Republic of Tanzania

**Keywords:** qPCR, Pooling, Community-wide molecular diagnostics, *Plasmodium falciparum* surveillance, Malaria elimination program

## Abstract

**Background:**

Molecular detection of low-density *Plasmodium falciparum* infections is essential for surveillance studies conducted to inform malaria control strategies in close-to-elimination settings. Molecular monitoring of residual malaria infections usually requires a large study size, therefore sampling and diagnostic processes need to be economical and optimized for high-throughput. A method comparison was undertaken to identify the most efficient diagnostic procedure for processing large collections of community samples with optimal test sensitivity, simplicity, and minimal costs.

**Methods:**

In a reactive case detection study conducted on Zanzibar, parasitaemia of 4590 individuals of all ages was investigated by a highly sensitive quantitative (q) PCR that targets multiple *var* gene copies per parasite genome. To reduce cost, a first round of positivity screening was performed on pools of dried blood spots from five individuals. Ten cycles of a pre-PCR were performed directly on the filter paper punches, followed by qPCR. In a second round, samples of positive pools were individually analysed by pre-PCR and qPCR.

**Results:**

Prevalence in household members and neighbors of index cases was 1.7% (78/4590) with a geometric mean parasite density of 58 parasites/µl blood. Using qPCR as gold standard, diagnostic sensitivity of rapid diagnostic tests (RDTs) was 37% (29/78). Infections positive by qPCR but negative by RDT had mean densities of 15 parasites/µl blood.

**Conclusion:**

The approach of pre-screening reactive case detection samples in pools of five was ideal for a low prevalence setting such as in Zanzibar. Performing direct PCR on filter paper punches saves substantial time and justifies the higher cost for a polymerase suitable for amplifying DNA directly from whole blood. Molecular monitoring in community samples provided a more accurate picture of infection prevalence, as it identified a potential reservoir of infection that was largely missed by RDT. The developed qPCR-based methodology for screening large sample sets represents primarily a research tool that should inform the design of malaria elimination strategies. It may also prove beneficial for diagnostic tasks in surveillance-response activities.

## Background

Surveillance is a key component of malaria control and elimination programmes [1]. Surveillance and response approaches require specific adaptation to the epidemiological and operational setting in which they are implemented. Depending on the exact objective of the surveillance activities, suitable data collection strategies and diagnostic tools may vary. A key interest is to understand population prevalence as a proxy for the transmission reservoir and transmission potential in a certain area. A number of publications have presented mathematical models and gametocyte data from endemic communities [2–6], all concurring in that, in addition to symptomatic malaria cases, also individuals with asymptomatic as well as submicroscopic infections contribute to the infectious reservoir. This silent reservoir is currently under investigation in many malaria endemic countries, and malaria elimination programmes aim to uncover and potentially track this source of transmission. To understand the relative importance of submicroscopic infections, their prevalence and densities are investigated in community samples of low parasite density. This is feasible by using molecular diagnostic methods. These methods are employed with the expectation that such data from the community may help to investigate residual transmission in close-to-elimination settings.

At low endemicity, malaria is known to cluster geographically and the exposure of individuals to malaria infection may vary substantially within a village and over time [7–9]. To capture such heterogeneity in case distribution, asymptomatic malaria cases are identified in a reactive case detection (RCD) approach [10, 11]. RCD is triggered by patients reporting to a health facility and diagnosed with malaria, based on laboratory confirmation. These index cases prompt a visit to the household of the patient (and sometimes to neighboring households) to identify additional malaria infections, most of which are expected to be asymptomatic [12]. The RCD strategy generally entails a targeted response, such as treating individuals of identified transmission foci. Thus, implementation of RCD aids in the containment of local epidemics of malaria and may help to control onward transmission from imported infections. Modeling indicated that RCD seems to be a promising approach to control residual malaria by complementing non-targeted interventions with targeting additional interventions or to support elimination in areas where the transmission potential is very low [13].

In pre-elimination settings, such as the site of this study in Zanzibar, passive case surveillance and elimination strategies further struggle with the fact that symptomatic cases are rare. On the other hand, active and reactive case detection include asymptomatic individuals, but parasite densities tend to be very low and difficult to detect with routinely applied diagnostic tools such as RDTs or light microscopy (LM). Meta-analyses comparing prevalence rates determined by PCR versus LM have demonstrated that the proportion of submicroscopic *P. falciparum* infections in community samples substantially increases with declining malaria transmission intensity [14, 15]. This trend was confirmed in the past few years by numerous molecular-epidemiological studies [15–21].

An extensive subpatent reservoir of malaria infections has major consequences for malaria surveillance activities, in particular in pre-elimination settings, where the aim is to interrupt local transmission. Recent data from a pre-elimination setting in Zambia showed that almost half of all infections remained undetected by RDT [22]. About a quarter of these infections were subpatent by RDT but carried gametocytes. To measure the magnitude of the infection reservoir missed in household surveys by employing RDTs, the current diagnostic tool for RCD, we undertook a molecular-epidemiological study in a close-to-elimination setting on Zanzibar. This study was nested into a larger project (Reactive Case Detection in Zanzibar: System Effectiveness and Cost, RADZEC) to evaluate the effectiveness of RCD in Zanzibar. Study details and epidemiological results were presented elsewhere ([23]; Stuck et al. pers. commun.). The focus of this report is the development and evaluation of an efficient diagnostic strategy for large numbers of samples collected during RCD in a pre-elimination setting. As samples collected from asymptomatic carriers in such settings mostly harbour low parasite densities, high diagnostic sensitivity and high throughput was the priority. In a low transmission setting with only few infected individuals in the community, molecular-epidemiological studies require screening of a large number of samples with high diagnostic sensitivity to identify the remaining infected individuals. A reduction of work load may be achieved by a sample pooling strategy prior to molecular diagnostics [24, 25]. Applying a multi-target molecular test is a precondition for both, pooling of several samples without losing sensitivity, and detection of asymptomatic, low-density infections. A quantitative PCR (qPCR) that targets the *P.falciparum* *var *gene family (*var*ATS us-qPCR) was previously developed to permit pooling without losing sensitivity [26]. The aims of this study, therefore, were to identify a time-efficient strategy for pooling multiple DBS samples, to simplify DNA extraction, and to develop a diagnostic method with high sensitivity and robust quantitation in detecting submicroscopic parasitaemia.

## Methods

### Study design

Since 2008, the Zanzibar Malaria Elimination Programme (ZAMEP) has been implementing a RCD system [23, 27]. The RADZEC project represents a rolling cross-sectional survey, whereby field data collectors accompanied district malaria surveillance officers (DMSOs) during follow-up visits to the households of malaria index cases who were detected at health facilities and flagged up through an electronic malaria case notification system (index cases). After the DMSOs finished with their investigation of the index household and departed, study staff continued surveying the four nearest neighbors and 5 households along a 200-m transect drawn in a random direction away from the index case household. Details on the study design were described previously elsewhere ([23]; Stuck et al. personal communication). The samples used in this molecular analysis were collected between June 1, 2017 and August 13, 2018.

The molecular epidemiological study consisted of a subsample of 156 clusters from the full rolling cross sectional study, each representing a follow-up investigation of an index case on Pemba and Unguja islands of the Zanzibar archipelago, Tanzania. From a large sample collection, clusters were selected for molecular analyses which contained samples from at least seven households. Each cluster included members of the index case household, of the four nearest neighbouring households and five transect households. A total of 664 residents of index case households were included, as well as 1955 members of neighbouring and 1971 of transect households. The index cases themselves were not included as they already had received treatment at the health facility 1 to 2 days prior to the follow-up household visit.

### Sample collection, storage and transport

During the follow-up household visits, usually within 5 days after the reporting of an index case, capillary blood was collected by finger prick from all consenting household members and neighbors older than 3 months. A malaria RDT (SD BIOLINE Malaria Ag Pf/Pan (Abbott)) was performed for on-the-spot malaria diagnosis. The DMSO treated all RDT-positive individuals within the index household according to the national guidelines. Individuals testing positive in non-index households, in which the DMSO was not present, were referred to the nearest health facility. A 100–150 µl whole blood sample was directly added to a pre-folded Whatman 3MM filter paper. Blood spots were dried in the field and then packed in individual plastic bags with desiccant and humidity indicator papers. Dried blood spots (DBS) were unpacked and re-dried after transport to the study office, re-packed with desiccant and humidity indicator paper, sealed in a zip-lock plastic bag and thereafter stored at room temperature.

### Preparation of dried blood spots with known parasite densities

For assay development and optimization, *P. falciparum* strain 3D7 was cultured in vitro and parasitaemia was determined by microscopy. To mimic an infected blood sample, 3D7 culture was diluted in malaria-negative whole blood to parasite densities ranging from 0.05 parasites/µl to 10^4^ parasites/µl. Negative controls consisted of whole blood from an uninfected volunteer. To simulate the conditions of DBS collected in the RADZEC study, blood of this volunteer was spotted in 50 µl aliquots onto Whatman 3MM filter paper, air dried overnight and stored at room temperature in plastic bags with desiccants.

### Comparison of DNA extraction by chelex, glassmilk, boil-and-spin to direct pre-PCR

For DNA extraction, five 3 mm discs were punched from the DBS using a hand-held paper craft punch. *var*ATS qPCR (details described below) was used to compare four different DNA extraction methods for DBS: (i) Chelex extraction was performed according to Plowe and co-workers, with minor modifications, such as two washing cycles with PBS instead of one, and omitting an incubation step in PBS for 15 min during PBS wash [28]. (ii) For boil-and-spin extraction, punches were incubated over night at 4 °C in 0.5% saponin/phosphate buffered saline (PBS). Saponin was removed after incubation and punches were washed twice by adding 1 ml of PBS, inverting tubes several times, spinning down briefly and removing PBS. Punches were transferred into a clean 1.5 ml Eppendorf tube and centrifuged briefly to collect and remove any remaining liquid. 50 µl ddH2O was added and samples were boiled at 95 °C for 30 min. Tubes were centrifuged for 5 min at 14,000 rpm. 25 µl of DNA solution was transferred into a new tube. (iii) Boil-and- spin plus glassmilk purification: After boil-and-spin extraction, DNA was purified using glassmilk (MP Biomedicals) according to the supplier’s instructions. (iv) Direct amplification of *P. falciparum* DNA in pre-PCR (details of final protocol described below) did not require any processing of punches from DBS. Four alternative polymerases for direct pre-PCR were tested during assay optimization: Phusion Blood Polymerase (Thermo Fisher Scientific), 2X KAPA HiFi HotStart (KAPA Biosystem), Hemo KlenTaq Polymerase (NEB), MyTaq DNA Polymerase (Bioline). Protocols and test results are provided in Additional file 1: Tables S1–S10. Experiments were performed at Swiss TPH, Basel, Switzerland.

### Molecular diagnosis in community samples

The bulk of samples to be analysed originated from RDT-negative household members. For detection of *P. falciparum* infections, samples collected from RDT-negative individuals were screened by pre-PCR/qPCR using a two-step process. First, pools of five samples were screened using one 3 mm punch per sample (one punch is equivalent to 3–4 µl of whole blood). In a second step, samples from positive pools were screened individually using five 3 mm punches per sample (equal to 15 µl of whole blood). Samples from RDT-positive household members and from index cases were processed separate from RDT-negative individuals and were directly screened individually in pre-PCR/qPCR using five 3 mm punches.

#### Direct-on-DBS pre-PCR

*P.falciparum* DNA was amplified directly from DBS punches using Phusion Blood Direct PCR Kit (Thermo Fisher Scientific). Primers targeted the conserved C-terminal region of the multi-copy *var* gene family [26]. A 55 µl reaction contained 1× Phusion Blood Buffer supplemented with 150 µM nucleotides and 450 nM forward and reverse primers (final concentration), 1 µl Phusion Blood Polymerase and five 3-mm punches. Cycling conditions were 98 °C for 5 min, followed by 10 cycles of 98 °C for 15 s, 55 °C for 30 s and 72 °C for 30 s. Pre-amplified PCR products were diluted 1:50 and used as template in *var*ATS qPCR.

#### Quantitative PCR

*var*ATS qPCR was performed using 4 µl of diluted pre-amplification product or DNA extracted by various approaches during comparison of extraction methods. The 12 µl reaction contained 1× GoTaq Probe Mastermix (Promega), 833 nM forward and reverse primer, and 416 nM probe (final concentrations). Cycling conditions in an ABI StepOne System were 95 °C for 2 min, 45 cycles of 95 °C for 15 s and 55 °C for 1 min. Parasite density was determined using a tenfold dilution row of the WHO 1^st^ international standard for *P. falciparum* DNA Nucleic Amplification Techniques (NIBSC), ranging from 4880 to 0.048 parasites/µl (the two lowest concentrations were run in duplicate). For direct-on-DBS pre-PCR followed by qPCR, this DNA standard was included in qPCR analysis starting from the pre-amplification step. In order to ensure equal amplification conditions of standard DNA and DBS samples, one 3-mm punch from a malaria-negative DBS was added to the pre-amplification reactions containing the standard DNA dilution. Two types of negative controls were used for all analyses: (i) parasite-negative DBS processed alongside samples through all extraction and amplification procedures, and (ii), 4 µl ddH_2_O added as blank to qPCR mix.

### Reproducibility, limit of detection (LOD), introduction of cut-off of parasite quantification by pre-PCR/qPCR

#### Reproducibility

Intra- and inter-assay variance, assessed by calculating the coefficient of variation (CV) for Ct values (expressed in %) were determined by testing replicate DNA dilution rows of the WHO 1st international standard for *P. falciparum* DNA Nucleic Amplification Techniques (NIBSC), supplemented with negative DBS punches as described above. Intra-assay variation was determined using 5 replicates of eight serially diluted samples (corresponding to 48,800, 4880, 488, 48.8, 4.88, 2.44, 0.488, and 0.244 parasites/µl), supplemented with 5 negative DBS punches, within a single pre-PCR/qPCR run. Inter-assay variation was determined using six serially diluted samples (corresponding to 4880, 488, 48.8, 4.88, 0.488, and 0.048 parasites/µl), supplemented with one negative DBS punch, in 28 separate pre-PCR/qPCR runs.

#### Limit of detection (LOD)

**Fig. 1:**
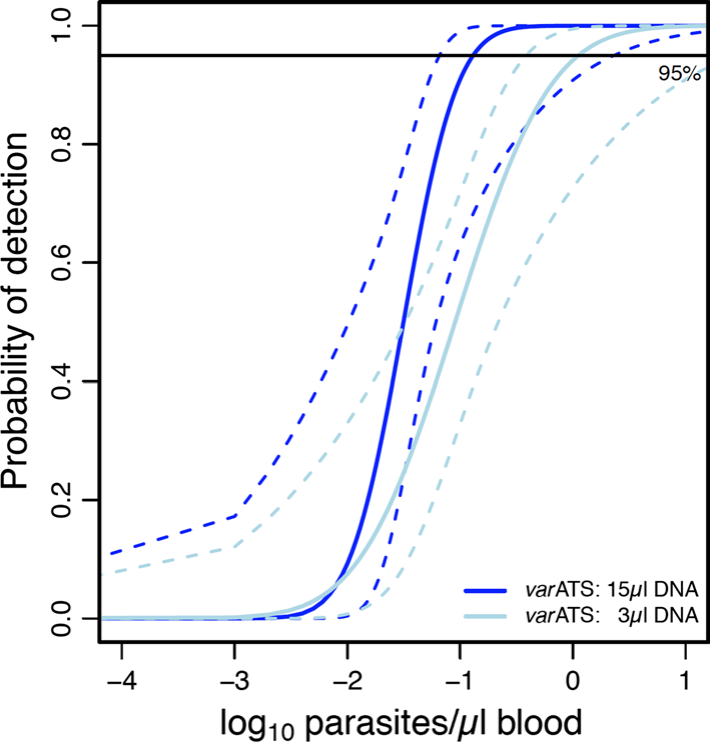
Limit of detection of *var*ATS qPCR determined by probit analysis. Based on a serial dilution of WHO standard material using either 3 µl of DNA supplemented by one negative DBS punch (representing LOD using 1 punch per DBS sample, light blue) or 15 µl of DNA supplemented with five negative DBS punches (representing LOD using 5 punches per DBS sample, dark blue)

The LOD was determined separately for the two PCR rounds: (i) screening of sample pools (one 3 mm DBS punch per sample in the pool) and (ii) testing of individual samples (using five 3 mm DBS punches). For this purpose, serial dilutions of the WHO DNA standard (NIBSC) were made and combined with punches from DBS impregnated with uninfected human whole blood. Pre-PCR and qPCR were performed in quintuple on 12 dilutions (corresponding to 48,800, 4880, 488, 48.8, 4.88, 2.44, 0.488, 0.244, 0.122, 0.048, 0.024, and 0.0048 parasites/µl). The LOD was determined using 3 µl and 15 µl of DNA, supplemented with 1 and 5 negative DBS, respectively, which represents the LODs using 1 and 5 punches from field sample DBS. A probit model was used to produce a regression line based on experimental replicates of the dilution series. The LOD was 1.12 parasites/µl for 3 µl of DNA (95 CI [0.39–27.81]) and 0.13 parasites/µl (CI95 [0.07–2.32]) for 15 µl DNA (Fig. 1). Hence, using five-fold more template material increased sensitivity 8.6-fold.

#### Cut-off setting

Since qPCR positivity for a sample of low parasitaemia strongly depends on a stochastic distribution of scarce parasites on the DBS, a positivity cut-off based on the LOD was introduced. The selected LOD of 0.13 parasites/µl permits detection of a parasite infection with 95% sensitivity when pools of 5 punches per DBS are analysed. This cut-off value for positivity was applied for all diagnostic assays performed on community samples.

## Results

### Comparison of extraction/amplification methods for DBS

**Fig. 2:**
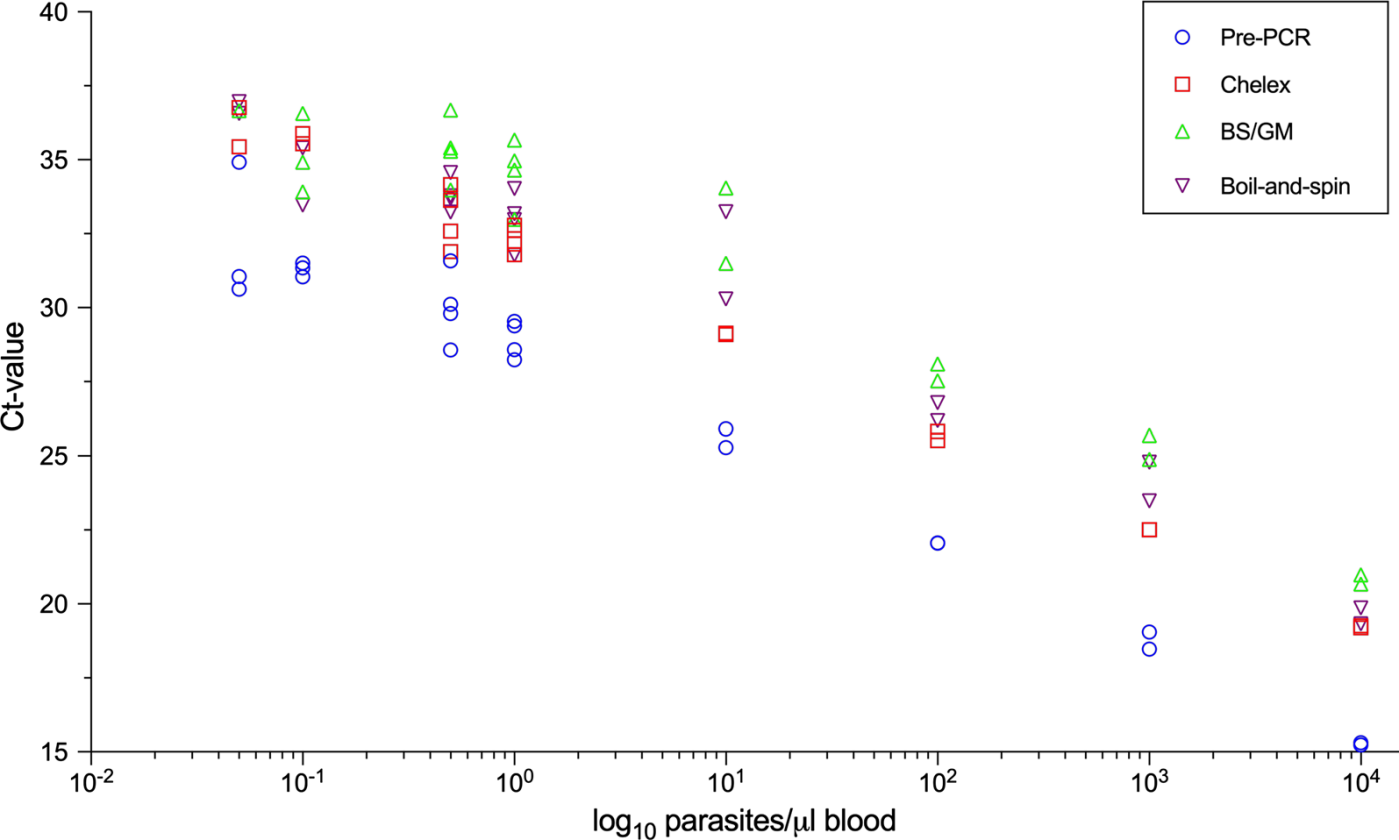
Comparison of Ct-values obtained by 4 DNA extraction methods in a *P. falciparum* dilution row spotted on DBS. Direct pre-PCR (blue), chelex extraction (red), boil and spin extraction followed by glassmilk purification (green) and boil and spin extraction (purple). Experimental replicates are represented by the same symbol

In search of the best suitable protocol for processing pooled DBS from large community surveys, the most sensitive and time-efficient combination of extraction and amplification was identified. To this end, test samples, ranging from 10^4^ parasites/µl to 0.05 parasites/µl, were analysed by chelex extraction, boil-and-spin extraction, boil-and-spin extraction followed by glassmilk purification, and pre-PCR performed directly on DBS punches (direct pre-PCR) (Fig. 2) followed by* var*ATS qPCR. Direct pre-PCR was the most sensitive method, giving positive results in 3/4 replicates of very low parasite densities (0.1 parasites/µl–0.05 parasites/µl) with least variation in measured densities between replicates. The direct pre-PCR also represents the least laborious method, as it requires no processing of DBS before PCR analysis. The second- and third- best methods were chelex extraction and boil-and-spin extraction, respectively. The least sensitive method was boil-and-spin followed by glassmilk purification. Because direct pre-PCR proved the fastest and most sensitive of all tested methods, this approach was chosen as standard procedure for the RADZEC RCD samples.

### Validation of parasite quantification

Prior to analysing DBS from study participants, parasite quantification was validated when performing direct-on-DBS pre-PCR. A tenfold dilution row of 3D7 parasites in whole blood spotted onto filter paper was analysed in parallel to a tenfold dilution row of the WHO 1st international standard for *P. falciparum* DNA Nucleic Amplification Techniques (NIBSC). To ensure that dilutions of WHO DNA standard and of 3D7 parasites were amplified under the same conditions, DBS by supplementing the WHO standard dilutions with DBS impregnated with uninfected blood for the pre-PCR step were mimicked. This was necessary, as pilot experiments had shown that the Phusion mastermix amplified purified DNA more efficiently when a negative DBS was added to the mastermix than purified DNA alone (Additional file 1: Figs. S1 and S2). This may be due to an optimization of Phusion direct blood kit specifically for DNA amplification in the presence of blood. Our experiments showed a qPCR efficiency of only 83% when the WHO standard DNA alone was pre-amplified in Phusion mastermix. In contrast, efficiency was 94% for DBS punches carrying the 3D7 culture dilution. Addition of a *P. falciparum*-negative DBS punch to pre-PCRs with WHO standard DNA restored qPCR efficiency to 100% (Additional file 1: Fig. S1).

To assess the reproducibility of quantification, intra- and inter-assay CV was determined by serial dilutions of the WHO standard DNA (Table 1A and B). Intra-assay CV of replicates ranged between 0.39 and 5.65% for different dilutions. These experiments used serially diluted DNA samples (in quintuplicate, supplemented with negative DBS) using 3 µl of DNA. When 15 µl of DNA were used, intra-assay CV ranged from 0.64 to 2.49%. Results were equally reproducible between runs, with inter-assay CV ranging between 0.93 and 3.49% for the different dilutions.

**Table 1:** Reproducibility of *P. falciparum* quantification by qPCR

A						
Parasites/µl	Mean (Ct)		SD		%CV	
	3 µl	15 µl	3 µl	15 µl	3 µl	15 µl
48,800	19.19	18.05	0.15	0.45	0.81	2.49
4880	20.63	19.41	0.06	0.25	0.28	1.26
488	24.34	22.68	0.12	0.24	0.50	1.06
48.8	27.58	25.95	0.12	0.17	0.45	0.64
4.88	31.12	29.76	0.12	0.25	0.39	0.83
2.44	31.91	30.40	0.63	0.49	1.99	1.62
0.48	35.61	32.83	2.01	0.46	5.65	1.39
0.24	34.98	33.78	1.12	0.98	3.20	2.89

B			
Parasites/µl	Mean (Ct)	SD	%CV
4880	20.39	0.22	1.07
488	23.72	0.26	1.09
48.8	27.17	0.25	0.93
4.88	30.47	0.38	1.25
0.488	33.35	0.9	2.68
0.048	34.76	1.21	3.49

A. Intra*-*assay variation of *var*ATS standard curve determined *via* Ct*-*values of eight serially diluted DNA samples (in quintuplicate, supplemented with negative DBS) using either 3 µl of DNA (equal to one punch per DBS) or 15 µl of DNA (equal to five punches per DBS)

B. Inter*-*assay variation of *var*ATS standard curve determined *via* Ct*-*values of six serially diluted DNA samples (supplemented by negative DBS) using 3 µl of DNA (equal to one punch per DBS) in 28 replicates

### Sensitivity after applying a robust density cut-off

Because many study participants carried very low parasite densities, we tested the reproducibility of parasite detection specifically in low-density field samples by repeating molecular diagnosis in independent replicates. Starting from the original DBS and using 5 DBS punches per sample, triplicates were analysed for 10 field samples with less than 0.1 parasites/µl, thus all representing samples that were positive but with densities below the cut-off set at 0.13 parasites/µl. Positivity for all 10 samples in at least one of the triplicates was confirmed. For 6/10 samples at least 2 replicates were positive. These results confirm that such very low-density infections are true positives and emphasize how much the detection of such infections by qPCR is determined by stochastic distribution of template in the starting material. Despite the repeated detection of low-positives with densities below 0.1 parasites/µl, a cut-off for qPCR-positivity at 0.13 parasites/µl was introduced, i.e. the LOD at which samples are detected with 95% probability. This cut-off may exclude some very low positive samples.

The stochastic distribution of parasite in samples with very low densities concentrations also affected our sample screening strategy: samples were first screened in pools of 5 samples with 1 DBS punch per sample, while the follow-up screens were performed on individual samples using 5 DBS punches, the latter aimed at reducing the stochastic effects. To understand the consequences of the stochastic parasite distribution when pooling 5 field samples for initial screening, we assessed the proportion of samples missed by this pooling strategy. For this purpose, 48 pools were randomly selected, corresponding to 240 individual samples, which all had been qPCR-negative in the first screening round. These 240 originally negative samples were screened individually using five punches per DBS. Before applying the cut-off for positivity, 18 low-positive individual samples (7.5%; 18/240) we found with densities of 0.01 parasites/µl–2.03 parasites/µl. When applying the chosen cut-off of 0.13 parasites/µl, 10 low-positive individuals (4.2%; 10/240) with densities of 0.13–2.03 parasites/µl remained positive and 8 individuals were below the cut-off of 0.13 parasites/µl and to be considered negative. Thus, in this small subset of samples, we showed empirically a loss in positivity by both, the cut-off for density and the stochastic distribution of low parasite densities on the DBS. Nevertheless, to maintain consistency in the database, all 18 positive samples newly identified in this additional experiment performed with in 48 originally negative pools were recorded negative in the final database as per protocol for community screening and analysis.

### Quality assurance for processing DBS from areas of very low prevalence and density

In order to validate the risk of cross-contamination on pre-PCR/qPCR plates, the following was repeated: (i) 22 samples with densities below 8 parasites/µl derived from RDT-positive household members of symptomatic index cases. These were repeated (in triplicate) because all samples from RDT-positive individuals, some of which with potentially very high parasite densities representing a risk for cross-contamination, were screened on a common PCR plate. All 22 low-positive samples were confirmed in independent repeat analyses. (ii) Molecular diagnostics on 7 RDT-negative field samples that had densities below 2 parasites/µl and had been identified in pools that contained a high-positive sample. Positivity was confirmed for all these samples. No further replicates were performed because of limited sample material.

### Prevalence of infection in community members from 156 clusters

Direct-on-DBS pre-PCR was performed on samples from 4590 individuals derived from 156 clusters, each triggered by an index case. 664 individuals belonged to the households of index cases, the remainder belonged to neighbouring and transect houses. By RDT, 0.7% of all individuals were *P. falciparum*-positive. Positivity by qPCR was 1.7% (Table 2). Of 33 RDT-positive individuals, 29 were confirmed positive by qPCR. RDT detected 37% (29/78) of qPCR-positive samples.

**Table 2:** Number of study participants that were *P. falciparum*-positive or -negative by RDT and qPCR

	RDT result	qPCR result
Negative	99.3% (4557/4590)	98.3% (4512/4590)
Positive	0.7% (33/4590)	1.7% (78/4590)

**Fig. 3:**
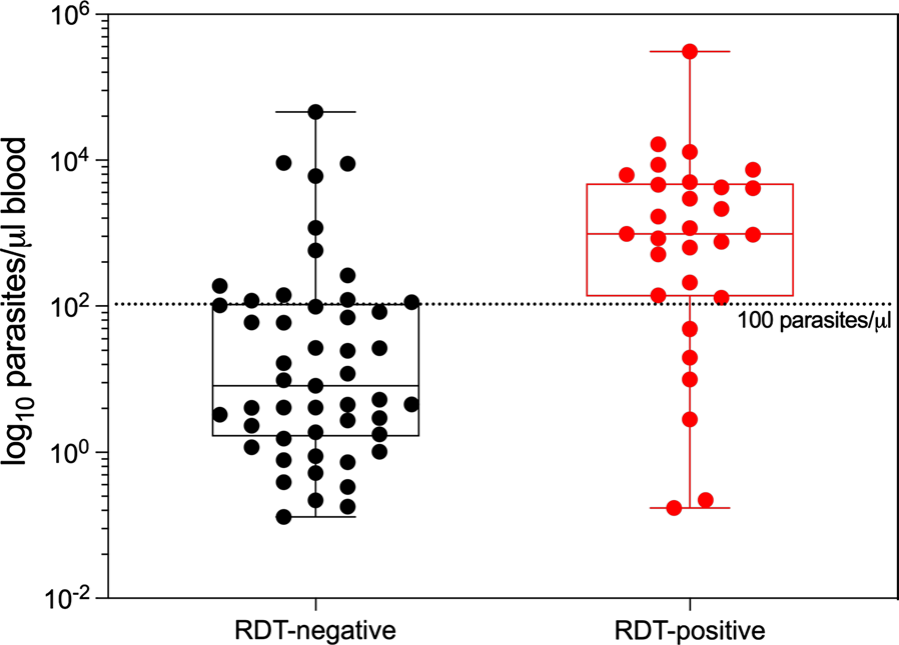
Proportion of *P. falciparum* density in RDT negative and RDT positive individuals detected by qPCR. Boxplot with median and IQR. Dotted line indicates parasite concentration 100 parasites/µl blood

The sensitivity of RDT compared to qPCR as gold standard was 37.2%, while the specificity was 99.9% (Table 3). Most individuals with a *P. falciparum* density below 100 parasites/µl were RDT-negative, whereas the majority of those with a density of 100 parasites/µl or higher were RDT-positive (Fig. 3).

**Table 3:** Comparison of RDT with qPCR results

	RDT negative	RDT positive	Total
qPCR negative			
Frequency	4508	4	4512
Percentage	99.9	0.09	100
qPCR positive			
Frequency	49	29	78
Percentage	62.8	37.2	100
Total			
Frequency	4557	33	4590

## Discussion

Sensitive detection and accurate quantification of low-density *P. falciparum* infections from DBS has become increasingly important in the context of describing residual malaria transmission in close-to-elimination settings. In areas of highly heterogeneous transmission it is of interest to identify risk factors of residual infections and to understand the infectious reservoir in the population. To provide optimal protocols for large-scale molecular-epidemiological studies in a pre-elimination setting, a simple but sensitive method combining pooling, extraction and amplification was developed. This strategy consisted of direct pre-PCR of pooled 3-mm punches from DBS, followed by *var*ATS qPCR.

Despite using a single DBS punch in a pool of 5 samples, the tested method was able to reliably identify 1 parasite/µl blood in a dilution series where DBS were reconstituted with WHO parasite density standard mixed with uninfected blood. The ultra-sensitive qPCR assay applied in this study targets multiple copies of *P. falciparum* *var* genes. A higher number of templates per parasite contributes to robust results in low-density infections and pools of these [26]. Nonetheless, the uneven distribution of the parasites in blood spotted on DBS poses an additional limiting factor. Hence, the sensitivity of our method might be slightly overestimated, as the WHO density standard was added as a DNA solution directly into the PCR mastermix, thus more even distribution of templates compared to spotting parasites. This discrepancy cannot be bypassed, because the use of an international reference standard is indispensable for inter-laboratory comparisons. The level of sensitivity of our method complies with the malERA consultative group-recommended detection limit for malaria pre-elimination settings of 1 parasite/µl blood [29]. Another previously published pooling approach used chelex extraction followed by *cytochrome b* nested PCR and reported a 100% sensitivity to detect a single positive sample with a density of 100 parasites/µl blood in a pool of 5 samples, and a 80% sensitivity to detect an infection of 10 parasites/µl in pooled analysis [25]. Compared to this previous study, the limit of detection was substantially more sensitive with 95% sensitivity to detect 1.12 parasites/µl blood on a single positive DBS punch screened in pools of five punches, i.e. together with 4 negative DBS. As both studies use multi-target qPCRs, this difference seems to be due to the chelex extraction used in this previous study. The chelex protocol has the advantage of being low-cost, but its disadvantage consists in a dilution of DNA in a larger volume than the original volume of blood, thus, sensitivity is compromised. It should be emphasized that for standardization sake, we measured both, LOD and CV, using the WHO standard DNA mixed with negative blood on DBS. The difference between introducing DNA or parasites into a reaction is that DNA in solution is more evenly distributed than when all target copies were contained in parasites. Owing to multi-copy genomic targets of the *var*ATS qPCR assay, the values for LOD and CV could prove slightly less sensitive, if whole parasites were analysed.

The ultra-sensitive diagnostic assay used in this study targets the *P. falciparum* *var* genes. These species-specific middle-repetitive sequences are dispersed throughout the genome [26]. Other Plasmodium species also occur in the study area [16], but these were not investigated so far. qPCR assays of comparable sensitivity were not yet available. For *P. vivax*, qPCR [30] and LAMP [31] assays were developed that detect mitochondrial DNA, of which numerous concatenated copies exist per parasite [32]. Mining of the *P. vivax* genome for species-specific, repetitive sequences identified a non-coding subtelomeric repeat, Pvr47 [33], yet, the Pvr47 copy number per genome (n = 14) was not higher than that of mitochondrial DNA. To our knowledge, no middle-repetitive sequences of higher copy-number than mtDNA have been identified and validated so far for qPCR diagnosis of the other human *Plasmodium* species.

Other methods than qPCR could be employed for reactive case detection. Loop-mediated isothermal amplification (LAMP) was used in Zanzibar in earlier studies [34]. However, LAMP was less sensitive than qPCR for detection of asymptomatic low-density infections [34]. Other disadvantages of LAMP are high prize for commercial LAMP kits, false positive results often arising from home-made master mixes, and the lack of parasite quantification (own unpublished observations, [30]). In a previous study in febrile children from Tanzania, the performance of the us-qPCR assay used here was compared with that of conventional RDT or highly sensitive RDT [35]. This earlier study showed that us-qPCR was substantially more sensitive in detecting low-density infection in children suffering from non-malarial fevers.

The advantage of direct-on-DBS pre-PCR is that the time-consuming extraction and purification of DNA is omitted, such as an overnight incubation in saponin as required in the chelex extraction. Punching disks from DBS is the most time-consuming step in the processing of samples. All DNA extraction methods equally require this initial step. Substantial time is saved by performing a pre-PCR amplification instead of chelex extraction, which reduces the processing time for 80 samples from 2 to 1 day. Pooling of punches from several DBS permits analysis of even more blood samples within 1 day. Reduction of processing time by pre-PCR justifies higher costs incurring from the requirement for a Phusion Blood Direct PCR Kit. The price per sample for the direct-on-punch pre-PCR method is around 2.4 US$ compared to 0.03 US$ per sample for chelex extraction. Introduction of pre-PCR permitted to introduce the highest possible concentration of parasite DNA into the amplification reaction. All alternative methods tested would have introduced less template into the qPCR mix, as pre-PCR overcomes loss of DNA during the extraction process. The major disadvantage of performing pre-PCR directly on filter paper was its high potential for contamination. Ten cycles of pre-amplification directly on DBS harbors dangers, mainly because it requires transfer of amplified product from pre-PCR into a second reaction tube or plate for qPCR. Such an open tube system requires utmost care because amplicons can potentially be transferred via aerosols or spills to neighboring wells leading to false positive results. The risk of contamination increases with increasing parasite density on the DBS. This risk requires an upscale in safety measures and controls as well as an adequate laboratory set up. In this study, the risk for contamination was minimized by dedicated rooms for master mix preparation, punching of samples, handling of post amplification product, and final qPCR reaction setup. Importantly, surfaces were decontaminated by exposure to UV light and bleach prior to and after completion of pipetting. Several negative controls were included in pre-PCR as well as qPCR to monitor any contamination. Despite installing preventive measures to avoid contamination, occasionally a no-template-control turned out positive. This could derive from aerosols or pipetting error. In case of contamination, all samples analysed in that experiment were repeated starting from new DBS punches. One way to further minimize cross-contamination, also employed in this study, was to analyse pools with high parasite concentrations (including all cases of symptomatic malaria) separately from low-density samples.

A relevant consideration in a molecular-epidemiological study is that pooling of samples from several individuals trades off test-sensitivity against the potential for high-throughput processing. This is because pooling of DBS reduces sensitivity of parasite detection because of a smaller blood volume processed. Due to space limits in reaction tubes, only five punches of 3 mm diameter corresponding to 3 µl blood each could be processed using our method. When samples were analysed individually, all five punches derived from one DBS, whereas for analysis of sample pools, only one punch per DBS could be processed.

During evaluation of the difference in LOD between the analysis of one sample (5 punches per DBS) versus a pool of 5 samples (one punch each), 8.6-fold loss in sensitivity was observed for pooling compared to individual screening. The thus reduced sensitivity by processing pools is in the sensitivity range of current molecular diagnostic assays used for screening of community samples. Using the *var*ATS us-qPCR compensated partly for the loss of sensitivity through pooling.

For processing a large set of samples from low-transmission settings as in this study, performing an initial screen on sample pools was necessary. When evaluating the potential to miss samples through pooling, 7.5% (18/240) low-positive samples would be gained by individual analysis, 10/240 (4.2%) would be positive above the cut-off. These numbers highlight the limitations in large studies and reporting the potential for false negatives is relevant. However, in the context of investigating the extent of the asymptomatic parasitaemia in the community, there is no need to identify the full depth of the subclinical reservoir, as such very low-density infections are unlikely transmitted [36].

This equally applies to definition of a density cut-off. A cut-off for qPCR-positivity of 0.13 parasites/µl blood was introduced to compensate for the variance caused by stochastic distribution of scarce parasites. Using the cut-off of 0.13 parasites/µl leads to omission of all low positive samples that would not be detected with certainty of less than 95%. Although samples below this cut-off were detected in some independent replicates, a very robust data set was created with records of positive samples that would be reproduced if repeatedly analysed.

Earlier Mass Screening and Treatment (MSAT) campaigns relying on RDT-based or LM-based diagnosis have not produced convincing results: Studies in Burkina Faso and Zanzibar found no sustained effect on incidence of anti-malarial treatment of asymptomatic *P. falciparum* carriers after screening and treatment campaigns [37, 38]. A population-wide malaria testing and treatment with RDTs and artemether-lumefantrine in southern Zambia, an area with heterogeneous transmission, showed an overall modest impact on decreasing the malaria infection burden [39]. A recent study in Indonesia reported similar results; after two or three rounds of MSAT using microscopy, little or no impact on malaria incidence was found [40]. Such little effect on incidence and prevalence is likely due to the large proportion of missed low-density infections, which will sustain transmission despite treatment of RDT-positive infections. A recent study in Zambia, performed in a close-to-elimination setting, showed that almost half of all PCR-diagnosed infections remained undetected by RDT, and about a quarter of these RDT-negative infections carried gametocytes and, therefore, may be infectious to mosquitoes [22].

The results obtained in Zanzibar are in line with previous observations of additional detection of *P. falciparum* infections by PCR [16, 41, 42]. The absolute numbers of infections detected by performing us-qPCR were small, with 45 infections detected additionally to RDT-positive individuals in a total of 4590 blood samples screened. RDT detected only 29 of the 78 qPCR-positive individuals and had a diagnostic sensitivity of 37%. Thus, to inform targeted response interventions, such as focal testing and treatment, RDT alone might not be sufficiently sensitive. However, it remains to be shown by further epidemiological analyses of these data from the Zanzibar household surveys, whether both diagnostics reveal the same epidemiological patterns and risk factors for infection in the various household types. Performing molecular diagnostics in the framework of elimination research represents a relevant expansion into a not yet well characterized, potential reservoir of infection.

It has to be emphasized that detection of very low-density infections is not trivial, and their detection is not necessary in many malariological studies [43]. However, low-density infections are relevant in studies like this one, aiming at a better understanding of transmission patterns. Even though low-density infections are unlikely to be transmitted at the time point of sampling, they might be transmitted later in the course of the infection. Thus, recording low-density infections with parasite densities from 1 to 10 parasite/µl generates more accurate and meaningful prevalence data compared to RDT-based data [2, 44]. Applying molecular tools in elimination research is useful for better understanding transmission patterns and underlying transmission risk in residual transmission scenarios and for the design and evaluation of targeted interventions.

In contrast to research studies, only cheap and simple-to-use methods, such as LM or RDT, are generally available for routine surveillance. Although the developed approach simplifies malaria diagnosis from DBS and supports high throughput screening, molecular diagnostic for programmatic use and routine implementation does not seem realistic currently, mainly due to a lack of funding, capacity and appropriate laboratory set-up. On the other hand, molecular parasite detection is very useful as a research tool for gaining knowledge on foci of residual malaria or the silent reservoir of transmission, as well as for informing mathematical modelling.

## Conclusions

A qPCR-based pooling approach was developed and applied that allows high-throughput and ultra-sensitive detection of *P. falciparum* DNA from DBS. This diagnostic approach applies pre-PCR amplification, which circumvents DNA extraction and facilitates pooling of five samples, but at the same time increases the risk of contamination. Thus, a laboratory set-up dedicated to PCR work is essential. This approach is suitable to quantify low-density *P. falciparum* infections in research studies aiming to better understand residual transmission or to generate accurate prevalence data for intervention monitoring and guiding targeted interventions.

## Supplementary information

**Additional file 1.** Additional figures and tables.

## Data Availability

All data generated or analysed during this study are included in this published article or accompanying additional file. The full data set can be made available upon request to M. Hetzel.

## Abbreviations

CV: coefficient of variation
DBS: dried blood spot
LM: light microscopy
MCP: malaria control programme
MEEDS: Malaria Epidemic Early Detection System
PCR: polymerase chain reaction
Pf-*var*ATS: *P. falciparum* *var* gene acidic terminal sequence
qPCR: quantitative PCR
RADZEC: Reactive Case Detection in Zanzibar: Effectiveness and Cost
RCD: reactive case detection
RDT: rapid diagnostic test
ZAMEP: Zanzibar Malaria Elimination Programme
