# Supplementary material for: Molecular methods for tracking residual Plasmodium falciparum transmission in a close-to-elimination setting in Zanzibar
Source: Malar J. 2020 Jan 29;19:50. doi: 10.1186/s12936-020-3127-x (PMC6988349; doi:10.1186/s12936-020-3127-x)

Additional file 1

Table S1: Reaction mix for pre-PCR targeting *Pf* *var*ATS using KAPA Polymerase performed directly on DBS

| **Reagent** | **conc_stock_** | **conc_final_** | **vol (1rxn)** |
| --- | --- | --- | --- |
| 5x KAPA HiFi Buffer | 5x | 1x | 10 µl |
| KAPA dNTPs Mix | 10mM | 0.3mM | 1.5 µl |
| *var*ATS Primer Mix (f/r) | 10µM each | 0.45MµM | 2.5 µl |
| 1U/µl KAPA HiFi HotStart DNA Polymerase |  |  | 1µl |
| PCR grade H_2_O, aliquoted |  |  | 40 µl |
| Infected DBS punch (3mm) |  |  | 1 punch |
| Negative DBS punch (3mm) |  |  | 4 punches |
| Total reaction volume |  |  | 55 µl |

Table S2: Cycling Protocol of the pre-PCR targeting *Pf* *va*rATS using KAPA Polymerase

| **Temperature** | **Time** | **Cycles** |
| --- | --- | --- |
| 95°C | 3 minutes | 1x |
| 98°C | 20 seconds | 10x |
| 55°C | 15 seconds |  |
| 72°C | 30 seconds |  |
| 72°C | 1 minutes | 1x |

Table S3: Reaction mix for pre-PCR targeting *Pf* *var*ATS using Hemo KlenTaq Polymerase performed directly on DBS

| **Reagent** | **conc_stock_** | **conc_final_** | **vol (1rxn)** |
| --- | --- | --- | --- |
| 5x Hemo KlenTaq Reaction Buffer | 5x | 1x | 10 µl |
| dNTPs | 2mM | 0.3mM | 5 µl |
| *var*ATS Primer Mix (f/r) | 10µM each | 0.45MµM | 2.5 µl |
| Hemo KlenTaq Polymerase |  |  | 4µl |
| PCR grade H_2_O, aliquoted |  |  | 33.5 µl |
| Infected DBS punch (3mm) |  |  | 1 punch |
| Negative DBS punch (3mm) |  |  | 4 punches |
| Total reaction volume |  |  | 55 µl |

Table S4: Cycling Protocol of the pre-PCR targeting *Pf* varATS using Hemo KlenTaq Polymerase

| **Temperature** | **Time** | **Cycles** |
| --- | --- | --- |
| 95°C | 3 minutes | 1x |
| 95°C | 20 seconds | 10x |
| 55°C | 30 seconds |  |
| 68°C | 1 minutes |  |
| 68°C | 10 minutes | 1x |

Table S5: Reaction mix for pre-PCR targeting *Pf* *var*ATS using MyTaq Polymerase performed directly on DBS

| **Reagent** | **conc_stock_** | **conc_final_** | **vol (1rxn)** |
| --- | --- | --- | --- |
| MyTaq Blood-PCR Mix, 2x | 2x | 1x | 25 µl |
| *var*ATS Primer Mix (f/r) | 10µM each | 0.45MµM | 2.5 µl |
| PCR grade H_2_O, aliquoted |  |  | 27.5 µl |
| Infected DBS punch (3mm) |  |  | 1 punch |
| Negative DBS punch (3mm) |  |  | 4 punches |
| Total reaction volume |  |  | 55 µl |

Table S6: Cycling Protocol of the pre-PCR targeting *Pf* *va*rATS using MyTaq Polymerase

| **Temperature** | **Time** | **Cycles** |
| --- | --- | --- |
| 95°C | 3 minutes | 1x |
| 95°C | 15 seconds | 10x |
| 55°C | 30 seconds |  |
| 72°C | 45 seconds |  |

The performance in direct pre-PCR of Phusion Blood Direct polymerase (Thermo Fisher Scientific) was compared to that of other polymerases using a dilution row of malaria positive (3D7) whole blood equivalent of 3µl per sample (1x3mm punch). Kapa HotStart polymerase (Kapa Biosystems) is a high fidelity polymerase with the advantage of a lower cost compared to Phusion polymerase. Kapa polymerase reached a similar sensitivity, but showed greater variations between replicates compared to Phusion polymerase. Hemo KlenTaq polymerase showed a low sensitivity, detecting only parasitaemias of 0.5 parasites/µl and above. MyTaq Blood polymerase (Bioline) reached a sensitivity similar to Phusion polymerase; however, at the time tested it was more expensive compared to Phusion polymerase. Thus, Phusion polymerase was selected for further validation and comparison to extraction methods.

Table S7: Sensitivity of parasite detection achieved by the optimized direct pre-PCR protocol using Phusion polymerase and *var*ATS qPCR. For this experiment a malaria positive whole blood equivalent of 3µl per sample (1x3mm punch) and a malaria negative whole blood equivalent of 12µl (4x3mm punches) were used.

| **Parasite/µl** | **Cт 1** | **Cт 2** | **Cт 3** | **Cт 4** | **Cт Mean** | **Cт StDev** |
| --- | --- | --- | --- | --- | --- | --- |
| 10^4^ | 17.98 | 18.21 | Not done | | 18.10 | 0.12 |
| 10^3^ | 24.95 | 20.61 |  |  | 22.78 | 2.17 |
| 10^2^ | 23.96 | 24.20 |  |  | 24.08 | 0.12 |
| 10^1^ | 27.25 | 30.23 |  |  | 28.74 | 1.49 |
| 1 | 29.84 | 33.85 | 31.06 | 30.44 | 31.30 | 1.54 |
| 0.50 | Undetermined | 30.66 | 29.95 | 28.93 | 29.85 | 0.71 |
| 0.10 | Undetermined | Undetermined | 32.47 | Undetermined | 32.47 | 0.00 |
| 0.05 | Undetermined | 31.01 | 31.07 | Undetermined | 31.04 | 0.03 |

Table S8: Sensitivity of parasite detection achieved by the optimized direct pre-PCR protocol using Kapa polymerase and *var*ATS qPCR. For this experiment a malaria positive whole blood equivalent of 3µl per sample (1x3mm punch) and a malaria negative whole blood equivalent of 12µl (4x3mm punches) were used.

| **Parasite/µl** | **Cт 1** | **Cт 2** | **Cт 3** | **Cт 4** | **Cт Mean** | **Cт StDev** |
| --- | --- | --- | --- | --- | --- | --- |
| 104 | 17.02 | 17.26 | Not done | | 17.14 | 0.12 |
| 103 | 20.57 | 20.78 |  |  | 20.68 | 0.10 |
| 102 | 23.73 | 23.91 |  |  | 23.82 | 0.09 |
| 101 | 27.76 | 27.86 |  |  | 27.81 | 0.05 |
| 1 | 32.18 | 33.02 | 32.10 | 32.35 | 32.42 | 0.36 |
| 0.50 | 35.21 | 32.39 | Undetermined | 31.33 | 32.98 | 1.64 |
| 0.10 | Undetermined | 37.03 | 31.72 | Undetermined | 34.38 | 2.65 |
| 0.05 | Undetermined | Undetermined | Undetermined | 35.89 | 35.89 | 0.00 |

**Table S9**: **Sensitivity of parasite detection achieved by the optimized direct pre-PCR protocol using Hemo KlenTaq polymerase and *var*ATS qPCR.** For this experiment a malaria positive whole blood equivalent of 3µl per sample (1x3mm punch) and a malaria negative whole blood equivalent of 12µl (4x3mm punches) were used.

| **Parasite/µl** | **Cт 1** | **Cт 2** | **Cт 3** | **Cт 4** | **Cт Mean** | **Cт StDev** |
| --- | --- | --- | --- | --- | --- | --- |
| 10^4^ | 17.78 | 17.17 | Note done | | 17.48 | 0.30 |
| 10^3^ | 20.94 | 22.69 |  |  | 21.81 | 0.87 |
| 10^2^ | 22.61 | 22.32 |  |  | 22.46 | 0.14 |
| 10^1^ | 27.54 | 27.27 |  |  | 27.40 | 0.14 |
| 1 | 32.31 | Undetermined | Undetermined | 39.35 | 35.83 | 3.52 |
| 0.50 | Undetermined | 32.31 | Undetermined | Undetermined | 32.31 | 0.00 |
| 0.10 | Undetermined | Undetermined | Undetermined | Undetermined | Undet. | Undet. |
| 0.05 | Undetermined | Undetermined | Undetermined | Undetermined | Undet. | Undet. |

Table S10: Sensitivity of parasite detection achieved by the optimized direct pre-PCR protocol using MyTaq polymerase and *var*ATS qPCR. For this experiment a malaria positive whole blood equivalent of 3µl per sample (1x3mm punch) and a malaria negative whole blood equivalent of 12µl (4x3mm punches) were used.

| **Parasite/µl** | **Cт 1** | **Cт 2** | **Cт 3** | **Cт 4** | **Cт Mean** | **Cт StDev** |
| --- | --- | --- | --- | --- | --- | --- |
| 10^4^ | 18.23 | 18.72 | Note done | | 18.47 | 0.24 |
| 10^3^ | 21.76 | 22.29 |  |  | 22.03 | 0.26 |
| 10^2^ | 24.82 | 25.50 |  |  | 25.16 | 0.34 |
| 10^1^ | 28.87 | 27.84 |  |  | 28.35 | 0.51 |
| 1 | 33.73 | 29.28 | 31.87 | 32.01 | 31.72 | 1.59 |
| 0.50 | 31.04 | 33.01 | 30.42 | 30.57 | 31.26 | 1.04 |
| 0.10 | Undetermined | 36.07 | Undetermined | Undetermined | 36.07 | 0.00 |
| 0.05 | 36.36 | 34.12 | Undetermined | 32.58 | 34.35 | 1.55 |

Figure S1: *var*ATS qPCR amplification of 10-fold dilution row of the international WHO DNA standard for Pf without a parasite-negative blood punch added to pre-PCR. Table below graph shows y-intercept, R^2^, and efficiency of the corresponding standard row.

Figure S2: *va*rATS qPCR amplification of 10-fold dilution row of the international WHO DNA standard for Pf with a parasite-negative blood punch added to pre-PCR. Table below graph shows y-intercept, R^2^, and efficiency of the corresponding standard row.

| Y-intercept | R^2^ | Efficiency |
| --- | --- | --- |
| 32.56 | 0.998 | 100.14% |

| Y-intercept | R^2^ | Efficiency |
| --- | --- | --- |
| 35.56 | 0.958 | 82.77% |


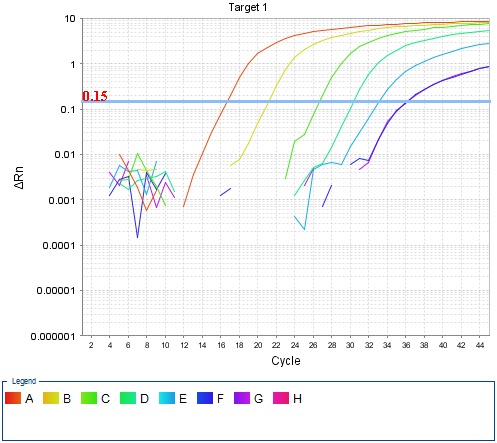

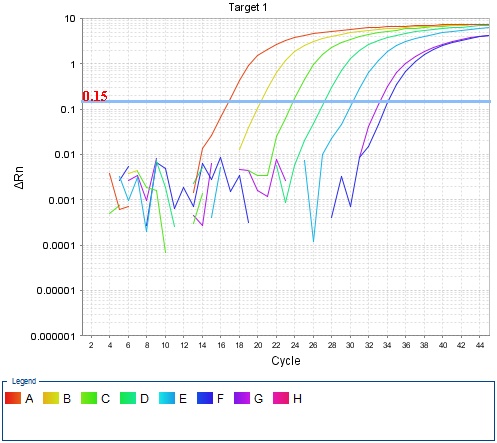

Supplement: Supplementary file 1 — Additional file 1. Additional figures and tables. [file 12936_2020_3127_MOESM1_ESM.docx]
